# Case Report: Case Series of Children With Multisystem Inflammatory Syndrome Following SARS-CoV-2 Infection in Switzerland

**DOI:** 10.3389/fped.2020.594127

**Published:** 2021-01-05

**Authors:** Athina Fouriki, Yves Fougère, Caroline De Camaret, Géraldine Blanchard Rohner, Serge Grazioli, Noémie Wagner, Christa Relly, Jana Pachlopnik Schmid, Johannes Trück, Lisa Kottanatu, Estefania Perez, Marie-Helene Perez, Damien Schaffner, Sandra Andrea Asner, Michael Hofer

**Affiliations:** ^1^Pediatric Immuno-Rheumatology of Western Switzerland, Department Women-Mother-Child, Lausanne University Hospital, Lausanne, Switzerland; ^2^Pediatric Immuno-Rheumatology, Department of Paediatrics, University Hospital, Geneva, Switzerland; ^3^Pediatric Infectious Diseases and Vaccinology Unit, Department Women-Mother-Child, Lausanne University Hospital, Lausanne, Switzerland; ^4^Pediatric Immunology and Vaccinology Unit, Faculty of Medicine, Geneva University Hospitals, Geneva, Switzerland; ^5^Division of Neonatal and Pediatric Intensive Care, Department of Paediatrics, Gynecology and Obstetrics, Geneva University Hospital, Geneva, Switzerland; ^6^Pediatric Infectious Diseases Unit, Department of Paediatrics, Gynecology and Obstetrics, Geneva University Hospital, Geneva, Switzerland; ^7^Division of Infectious Diseases and Hospital Epidemiology, University Children's Hospital Zurich, Zurich, Switzerland; ^8^Pediatric Immunology, University Children's Hospital Zurich, Zurich, Switzerland; ^9^Pediatric Infectious Diseases, Pediatric Institute of Southern Switzerland, Regional Hospital of Bellinzona, Bellinzona, Switzerland; ^10^Hôpital intercantonal de la Broye, Payerne, Switzerland; ^11^Pediatric Intensive Care Unit, Department Women-Mother-Child, Lausanne University Hospital, Lausanne, Switzerland; ^12^Pediatric Cardiology Unit, Department Women-Mother-Child, Lausanne University Hospital, Lausanne, Switzerland

**Keywords:** SARS-CoV-2, child, MIS-C, IL-1ra, Anakinra

## Abstract

Since the beginning of the severe SARS-CoV-2 pandemic, an increasing number of countries reported cases of a systemic hyperinflammatory condition defined as multi-system inflammatory syndrome in children (MIS-C). The clinical features of MIS-C can be an overlap of Kawasaki Disease (KD), Toxic Shock Syndrome (TSS), Macrophage Activation Syndrome (MAS), or have often an acute abdominal presentation. Intravenous immunoglobulin (IVIG) is recommended as first line therapy in KD. Recent evidence suggests intravenous immunoglobulins (IVIG) resistance in some cases of SARS-CoV-2 related MIS-C, thereby questioning the benefit of immunomodulators such as IL-1 or IL-6 blocking agents. We report on a cohort of 6 Swiss children with SARS-CoV2 related MIS-C presenting with clinical features compatible with Incomplete KD and Toxic Shock Syndrome associated to a cytokine storm. Serum cytokine profile investigations showed increased IL1RA levels (8 to 22-fold) in 5 of the 6 patients (one patient had not been tested), whereas, IL-6 serum levels were increased only in the 3 patients of the 6 who were tested. With exception of one patient who had only benefited by Anakinra, all patients received at least one dose of IVIG. One patient has only received Anakinra with favorable evolution, and three patients had also a steroid treatment. In addition to all this anti-inflammatory medication two patients have also received one dose of anti-IL6. In conclusion, our case series reports on clinical and laboratory findings of most of Swiss cases with MIS-C and suggests the use of Anakinra as an alternative to steroids in these children, most of whom presented with high IL-1RA levels.

## Introduction

Since the beginning of the severe acute respiratory syndrome coronavirus 2 (SARS-CoV-2) pandemic, an increasing number of countries reported cases of a systemic hyperinflammatory condition defined as multi-system inflammatory syndrome in children (MIS-C). This hyperinflammatory condition has also been termed as pediatric multisystem inflammatory syndrome (PIMS), pediatric inflammatory multisystem syndrome temporally associated with SARS-CoV-2 (PIM-TS), pediatric hyperinflammatory syndrome, or pediatric hyperinflammatory shock (1–3). Initially published case series from France (4), New-York (5), and England (6) included 35, 33, and 58 children, respectively, whereas further cases are being published (7, 8). Clinical description revealed from 13 of the 30 British children aged between 4 and 14 years referred to either warm vasoplegic shock or acute abdominal and sepsis-like presentations (2). Italian data published on May 2020 described incomplete Kawasaki Disease-like presentations in 5 of their 10 children aged between 3-16 years of age, six of these ten patients presented with coronary inflammation and aneurysms (7). Common observations delineated from this MIS-C referred to overlapping clinical features of Kawasaki Disease (KD), Toxic Shock Syndrome, and Macrophage Activation Syndrome with acute abdominal presentations upon admission (1, 2, 9–13). While intravenous immunoglobulin (IVIG) is recommended as first line therapy and is successfully used in KD, some refractory cases may benefit from treatment with an interleukin-1 receptor antagonist (Anakinra). Recent evidence suggests IVIG resistance in some cases of SARS-CoV-2 related MIS-C, thereby questioning the benefit of immunomodulators such as IL-1 or IL-6 blocking agents (7, 14). Switzerland reported over 31,000 persons detected positive to SARS-CoV-2, 3.2% were children (0-18 years old) (15). From these, 9 children had MIS-C and documented SARS-CoV-2 infection by PCR or serologies. We describe the clinical characteristics, laboratory data and treatment management of the collected 6 of these 9 Swiss children with MIS-C, the remaining 3 have already been reported in another paper (16).

## Methodology

Our clinical study is a case series that includes all eligible patients identified during the study registration period (consecutive, formal). It describes the experience on a small group of patients (observational, descriptive research design), contains demographic information about them and was conducted retrospectively. The patients were treated in the order in which they were identified, without a group control.

### Inclusion Criteria

All patients with a positive serology for SARS-CoV2 (IgG serology) and symptoms, signs, and laboratory markers in favor of a systemic hyperinflammatory condition. The patients were identified by their severe clinical presentation with need of hospitalization, the increased laboratory inflammatory markers and their positivity for SARS-CoV2.

### Exclusion Criteria

Patients with evidence of a hyperinflammatory state having negative serology for SARS-CoV2 and negative nasopharyngeal smear. Patients whose clinical presentation had already been published in another study, by the hospital center that followed them. One patient whose parents did not sign the consent about the publication.

### Centers Participated

Four hospitals of Switzerland participated in this study, including the University Hospital of Lausanne (CHUV), of Geneva (HUG), of Zurich (USZ) and the Regional Hospital of Bellinzona.

### Ethics/Consent

The parents of all patients included in our study were informed about the management of these hyperinflammatory conditions, including the different treatments. They signed a consent form for the participation of their children in the publication and about their medium and long-term follow-up.

### Quantifications of Cytokine Profile

The quantification of cytokines was made by a commercially available multiplex beads immunoassay, based on the Luminex platform (Magnetic Luminex® Performance Assay, R&D Systems, Minneapolis, USA) according to supplier's instructions. Briefly, beads conjugated to the analyte-specific capture antibodies, samples, standards and controls, were incubated at room temperature for 3 h. Biotinylated detector antibodies and R-phycoerythrin–conjugated streptavidin (SAPE) were subsequently added. The mean fluorescence intensity of each analyte was read on the Bio-Plex 200 array reader (Bio-Rad Laboratories) using the Luminex xMAP Technology (Luminex Corporation). Sample concentrations were calculated using a five-parameter logistic regression curve (Bio-Plex Manager 6.0). Interassay variation coefficients were monitored using internal controls. These were below 15% for all (17).

## Clinical Characteristics, Laboratory and Imaging Findings

Table 1 displays clinical characteristics, laboratory and imaging findings as well as treatments of all patients. Five of the 6 patients were males, with a median age of 10.5 years (Interquartile Range (IQR): 8.5-11 years). None presented any co-morbidity. Four patients were Caucasian, 1 African, and 1 Afro-Caucasian. None of the patients presented respiratory symptoms or fever suggestive of COVID-19 in the weeks (4–6 weeks) preceding their admission. However, family members from patient 1 and 2 presented respiratory symptoms consistent with COVID-19, a month prior to hospital admission, some family members of patient 4 had anosmia and mild respiratory symptoms three weeks prior to admission. SARS-CoV-2 was documented by real time (RT-PCR) collected from nasopharyngeal swabs (NPS) in 5 of the 6 patients. The PCR threshold cycle (TC) were >35 for E and N2 genes in patients 1 to 3, thereby supporting a low viral load. All patients presented a positive IgG serology (Roche serology) (18).

**Table 1:** Clinical and biological characteristics of Swiss children admitted in Hospital with MIS-C.

	**Patient 1**	**Patient 2**	**Patient 3**	**Patient 4**	**Patient 5**	**Patient 6**
**Clinical features**						
Age in years/sex	5/M	8/M	10/M	14/M	11/F	11/M
Ethnicity	Caucasian	African and Caucasian	African	Caucasian	Caucasian	Caucasian
BMI (kg/m^2^)/comorbidities^*^	17.02 (P:75-90)/none	16.52 (P:50-75)/none	18.9 (P:75-90)/none	24.8 (P:75-90)/none	Weight: 30 kg/none	18.5 (>P97)/none
**Presenting symptoms**						
Fever>4 days, >40°C	+	+	+	+	+	+
Abdominal pain	-	+	+	+	+	-
Diarrhea/Emesis	+/-	+/+	+/+	+/+	-/-	+/+
Rash	-	+	-	+	+	+
Conjunctival injection	+	-	-	+	+	+
Lymphadenopathy	+	-	-	-	-	-
Extremity edema/erythema	+/+	-/+	-/-	-/+	+/+	-/-
Headache/irritability	+/+	-/-	-/-	+/+	-/-	+/+
Phono photophobia/petechiae	+/+	-/-	-/-	+/+	-/-	-/-
Cheilitis	-	-	-	-	+	-
Respiratory insufficiency	+	+	+	+	+	+
Shock	-	+	+	+	+	+
Acute encephalopathy	-	+	-	-	-	+
**Cardiopulmonary support**						
Ventilation support	O_2_ support	MV	MV	MV	O_2_ support	MV
Vasoactive support/diuretics/corticosteroids	-/+/-	Mil-Dopa-NorAdre/-/-	Mil-Dopa-NorAdre-Dobut/+/-	Adre-NorAdre/-/-	NorAdre/-/-	Mil-Dopa-Adre-NorAdre-Vasopr/+/+
**Maximal values in laboratory**						
C-reactive protein (mg/L) (Ref: <10 mg/L)	>500	378	176	415	200	200
Procalcitonin (mcg/l) (Ref: <2 mcg/l)	1.67	6.4	30.2	4.16	10	50
Ferritin (mcg/L) (Ref: 14-101 mcg/L)	176	912	799	1,316	270	1,155
Neutrophil count (G/L) (Ref: 1.5–8.0 G/L)	8.3	28.42	15.94	17.69 (29.75 under steroids)	10	23
N-Terminal pro-Brain natriuretic peptide -proBNP (ng/L) (Ref: <145 ng/l)	11,383	42,225	10,894	7,206	2,808	>70,000
Interleukin 1 antagonist receptor (pg/ml) (Ref: ≤720 pg/ml)	2,215	14,273	16,680	Not tested	>5,000	>5,000
Interleukin 6 (pg/ml) (Ref: ≤11 pg/ml)	<11	<11	<11	396	90	516
Acute kidney insufficiency	-	-	-	-	+	-
Hepatic cytolysis	-	-	+	-	+	+
**SARS-CoV-2 testing**						
SARS-CoV-2 serology	+	+	+	+	+	+
**Imaging**						
Coronary dilation (Z-score)	+ (+4.25)	+(+2.9)	-	-	-	-
IVA aneurysm	+	-	-	-	-	-
Cardiac dysfunction	-	LVEF 45%	LVEF 45%	-	-	LVEF 45%, systolic and diastolic dysfunction of the left ventricle
Cardiopulmonary imaging (US; X-ray; C/T scan)	Mild pleural effusion	Bilateral broncho-pneumonia	Moderate pleural effusion	-	Mild pleural and pericardial effusion	Pleural effusion and thickening of the inter-lobular septas
Cerebral imaging (MRI)	Normal	-	-	Localized meningeal enhancement sulcus centralis and postcentral region(R), no angiopathy or thrombosis	-	Normal
ENMG	Not tested	Not tested	Not tested	Not tested	Not tested	Moderate motor-sensitive axonal polyneuropathy
EEG	Not tested	Not tested	Not tested	Not tested	Not tested	Moderate reactive encephalopathy with slight asymmetry against the right hemisphere
Abdominal imaging (US; C/T scanner)	-	Mesenteric adenitis, ascites, paralytic sub-ileus	Abdominal lymphadenopathy and ileitis	-	Abdominal lymphadenopathy	-
**Antibiotics (duration-days)**						
	Fclox (14)	Cftx+Met (10)	Amox/clav (5)	Cftx (4)	Cftx (3)+Clinda (4), Merop+Vanco (3)	Cftx (3)
**Immunomodulation–***Initiation of treatment* (Day 0 = admission to the hospital)						
Number of doses of IVIG (2 g/kg)	1	2	0	1	1	1
	Day 3	Day 2 + day 3		Day 2	Day 1	Day 1
Acetylsalicylic acid	+	+	-	+	+	+
Methylprednisolone or prednisone 2 mg/kg/d	-	-	-	+	+	+
				Day 3 (4 weeks in total)	Day 2 (9 days in total)	Day 1 (3 days in total)
Anakinra	-	2 mg/kg for 3 days, 4 mg/kg for >7 days—*Started on day 6*	2 mg/kg >7 days –*Started on day 3*	2 mg/kg for 10 days —*Started on day 5*	1.7 mg/kg for 5 days —*Started on day 4*	1.3 mg/kg 2 doses —*Started on day 2*
Tocilizumab	-	-	-	-	8 mg/kg 1 dose—*Started on day 3*	8 mg/kg 1 dose—*Started on day 3*
HCQ	-	-	-	-	-	5 mg/kg 1 dose—*Started on day 3*

*O_2_, oxygen; Dobut, dobutamine; ENMG, electroneuromyography; BMI, body mass index; MV, mechanical ventilation; NorAdre, noradrenaline; Mil, Milrinone; Vasopr, vasopressine; Dopa, dopamine; Adre, adrenaline; Vanco, vancomycin; Amox/clav, amoxicillin/acid clavulanic; Cftx, ceftriaxone; Clinda, clindamycin; Fclox, flucloxacillin; Mero, meropenem; Met, metronidazole; LVEF, left ventricle ejection function; IVIG, intravenous immunoglobulins; HCQ, hydroxychloroquine; MRI, magnetic resonance imaging; EEG, electroencephalography; US, ultrasonography; C/T scanner, computed tomography*.

*IMCU, Intermediate care unit; ICU, Intensive care unit; NPS, nasopharyngeal swab*.

* *Percentile source: WHO Growth Charts*.

All 6 patients presented with persistent fever above 39°C (more than 5 days) and were treated with broad spectrum intravenous antibiotics at admission, despite negative blood cultures. None presented with respiratory symptoms or infiltrates on chest X-rays suggestive of a lower-respiratory tract SARS-CoV-2 infection. All presented an important inflammatory syndrome with high levels of CRP (median: 289 mg/l, IQR: 200-406 mg/l), neutrophilia and increased blood levels IL-1 receptor antagonist (IL-1RA), except for patient 4, where IL-1RA serum levels were not measured. Patients 2, 3 and 6 presented a transitory myocardial dysfunction and patients 1 and 2 with a coronary artery dilatation at the admission. None of the patients had a persistent cardiac dysfunction upon discharge from the hospital. In addition to fever, patients 1 and 4 initially presented with meningitis-like symptoms; albeit with normal cerebrospinal fluid findings. Both children subsequently presented clinical signs, suggestive of Kawasaki Disease (KD), including persistent fever lasting over 5 days, bilateral adenopathy, palmar erythema, hand edema and bilateral conjunctival injection. In addition, patient 1 presented with a coronary dilation [z-score +4.25 (19)] with IVA aneurysm (aneurysm of the left interventricular coronary) on cardiac echography. Patients 2, 3, 5, and 6 presented an acute abdominal clinical picture, with hemodynamic instability requiring aminergic support. Patients 2 and 3 presented with an acute-appendicitis-like clinical picture. Patients 5 and 6 presented some acute abdominal symptoms associated with conjunctival injection and a rash, albeit not meeting all criteria for complete KD. Serum cytokine profile investigations showed increased IL1RA levels (8 to 22-fold) in 5 of the 6 patients (one patient had not been tested), whereas IL-6 serum levels were increased only in the 3 patients of the 6 who were tested. The rest of the cytokine profile was normal.

**Figure 1:**
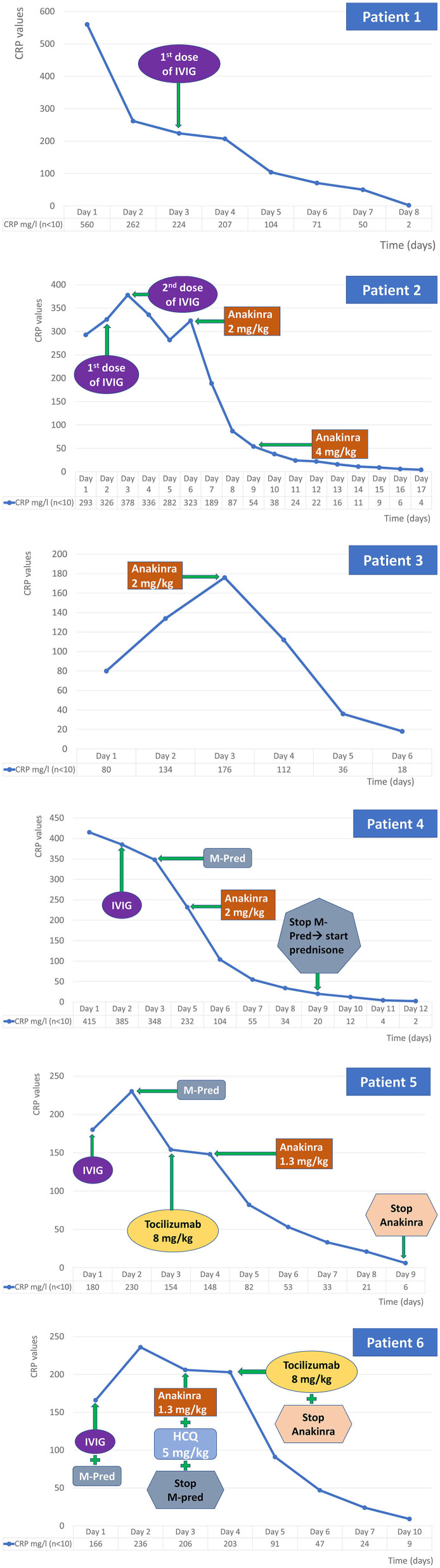
Chronology of the inflammatory markers and the treatments used. IVIG, intravenous immunoglobulins.

The treatments introduced for each patient and their CRP values are shown in Figure 1. With exception of patient 3, all patients received at least one dose of intravenous immunoglobulins (IVIG 2 g/kg). Patient 1 benefited of one dose of IVIG with a positive clinical and biological response. Patient 2 received a second dose of IVIG (the same dose as the first one administrated, 2 g/kg) 48 h apart (on day 3 of admission) and subcutaneous anakinra at 2 mg/kg/day for 3 days (started on day 6), increased up to 4 mg/kg/day given his persistent fever and increased inflammatory parameters. Patient 3 only received anakinra (2 mg/kg) with favorable clinical and biological response. Patients 4, 5, and 6 required one dose of IVIG followed by anakinra (2 mg/kg) and steroids for patient 4, which resulted in the resolution of inflammatory parameters. Patient 4 was initially under methylprednisolone (for one week) and then he benefited from a relay by prednisone with a tapering regimen over 3 weeks. In addition, patients 5 and 6 also received one dose of tocilizumab (8 mg/kg). Patient 5 received tocilizumab in addition to steroids which were started the day before, because of persisting fever and hemodynamic instability, with a good initial response. However, after 24 h, the patient presented again fever, diffuse myalgia, burning on palms of hand and feet and hemodynamic instability, in tandem with persistence of inflammatory markers. All these reasons have motivated the addition of Anakinra. Patient 6 was remaining sub febrile with a massive inflammatory syndrome, pejoration of the Pro-BNP, and cardiac echography showing an alteration of the cardiac function with suspicion of myocarditis, despite 2 doses of Anakinra and those parameters have motivated the addition of Tocilizumab one day after the introduction of Anakinra and hydroxychloroquine.

It is important to highlight that the results of the cytokines were not available when we considered to change treatments. Four patients (patient 1, 4, 5, and 6) were also treated with acetylsalicylic acid at anti-inflammatory doses.

## Discussion

We report on a cohort of Swiss children with SARS-CoV2 related MIS-C presenting with clinical features compatible with Incomplete KD and Toxic Shock Syndrome associated to a cytokine storm suggestive of a macrophage activation syndrome (MAS) without fulfilling the criteria for Hemophagocytic Lymphohistiocytosis (HLH). As already documented elsewhere (2, 4, 6, 7, 16, 20) and as described in 4 of our 6 patients, many children with MIS-C present some criteria for complete or incomplete KD, appendicitis-like abdominal symptoms and important hyperinflammatory syndrome. Important epidemiological differences between KD and MIS-C include the ethnicity and age of affected patients. While KD primarily affects infants and young children with a 20-fold increased incidence in Asian children (21, 22), MIS-C has been reported among older children, predominantly from an African and Hispanic ethnic background, but this is not always the case (21, 22). These findings could suggest an underlying specific host polymorphism triggering a cytokine cascade secondary to SARS-CoV-2 infection. Hospitalizations due to SARS-CoV-2 were higher in the same geographic areas in which we later discovered the cases of MIS-C, thereby reinforcing an association between MIS-C and SARS-CoV-2. The contribution of a specific SARS-CoV-2 strain is debated, as some of the European SARS-CoV-2 strains could harbor a mutation favoring TCR binding (23) thereby possibly explaining the occurrence of MIS-C in Western countries rather than in Asia. Yet, several different strains have been documented in Europe and in the US (24). As such, the pathophysiology of progression to MIS-C in specific children remains unclear. The contribution of a specific strain in our cohort of patients could not be documented given that genotyping analyses were limited due to the very low viral loads detection. MIS-C also differs from acute COVID-19 illness which tends to be most severe in infants <1 year of age (7, 25, 26). In addition, MIS-C is typically documented up to a month after the peak of SARS-CoV-2, thereby coinciding with the timing of acquired immunity and suggesting a post-infectious inflammatory reaction rather than an acute infectious process (7, 21, 22, 27).

The pathophysiology and the mechanisms by which SARS-CoV-2 triggers an abnormal immune response leading to MIS-C remain poorly understood. Current evidence reports a hyperinflammatory reaction presenting similarities with TSS, incomplete KD and MAS, suggesting activation of the innate immune response with massive pro-inflammatory production (28). SARS-CoV-2 may act as a superantigen (23) similarly to the staphylococcal enterotoxin B (SEB) known to bind to the costimulatory molecule CD28 and the T cell receptor (TCR), thus mediating TSS. The activation of the innate immunity during SARS-CoV-2 infection leads to a cytokine release syndrome named “cytokine storm” characterized by persistent fever and markedly elevated cytokines mostly TNF-α, IL-1β, IL-1RA, sIL-2Rα, IL-6, IL-10, IL-17, IL-18, IFN -γ, MCP-3, M-CSF, MIP-1a, G-CSF, IP-10, and MCP (29). Most of recent published case series of MIS-C patients documented increased IL-6 levels; (28, 30, 31) the serum levels of other pro-inflammatory cytokines being underreported, except for normal levels of IL-1ß in one case (28). Our case series differs in the documentation of very high serum interleukin-1 receptor antagonist (IL-1RA) levels, an important marker for innate immunity activation, among 5 patients; IL-6 serum levels being elevated in only 3 of our 6 patients. These findings may suggest that the activation of the IL-1 pathway also represents an important mechanism of activation in MIS-C as already documented in other conditions such as MAS, TSS, and KD (32). Given similarities between MIS-C and KD and the contribution of IVIG in TSS, all but one patient were first started on IVIG with or without corticosteroids (16, 21, 22). Three of our patients received either parenteral methylprednisolone or a short course of oral prednisone. Recent evidence reports on a one-third reduced deaths among intubated and ventilated adults with acute COVID-19 treated with dexamethasone for 10 days when compared with patients randomized to usual care alone (33). As such, the benefit of dexamethasone has mostly been suggested among adults with severe COVID-19 disease, although it might also be useful in MIS-C given its inhibition of cytokine production (33) In addition, glucocorticoids are also recommended for patients with KD with persistent fever after IVIG or coronary arteries dilatation and are considered among those with cytokine release syndrome. In this regard, the benefits of glucocorticoids as first-line treatment for inflammatory diseases remain undisputable, although biological agents are also attractive alternatives as reported from children with JIA (34). Several reports refer to the use of anakinra (an IL-1 receptor antagonist RA recombinant) and tocilizumab (an antibody against the IL-6 receptor) in patients infected by SARS-CoV-2 with hyperinflammatory state and in a minority of those with MIS-C in addition to IVIG and glucocorticoids (5, 20, 30, 35). Anakinra is specifically attractive due to its good safety profile and short half-life.

This case series displays a range of heterogeneous treatments, prescribed by different hospitals in Switzerland, which allows us to draw a first set of meaningful conclusions on the effectiveness of combinations of different drugs on patients infected by SARS-CoV-2. Specifically, only one patient (patient 1) received solely IVIG, and the remaining five received anakinra either alone or in combination with other drugs. From these five patients, one (patient 3) only received anakinra and the four others also received additional immunomodulators. Notably, one patient (patient 2) received solely IVIG as supplement treatment and the remaining three (patients 4,5 and 6) received steroids in addition to IVIG; two out of them (patients 5 and 6) also received one course of tocilizumab. All patients had a favorable response to these different treatments.

In conclusion two of our six patients were successfully treated with anakinra without receiving steroids or other immunosuppressive treatment. This observation combined with the documentation of increased IL-1RA levels in 5 of our 6 patients further supports the use of anakinra in children presenting a multi-system inflammatory syndrome (MIS-C). Concerning the other targeted treatments and notably the tocilizumab, it could be either an alternative or a supplementary treatment depending on the cytokine profile presented by the patient, which varies between individuals.

Despite the limited number of cases included in our study in addition to previous reports on 3 of the 9 Swiss MIS-C, we believe that we add on current published evidence by providing a complete cytokine profile from most of the included cases and provide some evidence on the use of anakinra in MIS-C. This type of study is most useful for describing the potential effectiveness of new interventions and for describing the effectiveness of interventions on unusual diagnoses.

In conclusion, our case series reports on clinical and laboratory findings of most of Swiss cases with MIS-C and suggests the use of anakinra as effective treatment, especially in children with documented high IL-1RA levels.

## Data Availability Statement

The original contributions presented in the study are included in the article/supplementary material, further inquiries can be directed to the corresponding author/s.

## Ethics Statement

The studies involving human participants were reviewed and approved by Commission cantonale (VD) d'éthique de la recherche sur l'être humain. Written informed consent to participate in this study was provided by the participants' legal guardian/next of kin. Written informed consent was obtained from the individual(s), and minor(s)' legal guardian/next of kin, for the publication of any potentially identifiable images or data included in this article.

## Author Contributions

AF, YF, SA, and MH were involved in the conception of the paper. AF, YF, CD, SA, and MH analyzed the cases. AF wrote the first draft of the manuscript. AF, YF, and SA wrote sections of the manuscript. All authors contributed to manuscript revision, read, and approved the submitted version.

## Conflict of Interest

The authors declare that the research was conducted in the absence of any commercial or financial relationships that could be construed as a potential conflict of interest.
